# pH-mediated potentiation of gallium nitrate against *Pseudomonas aeruginosa*

**DOI:** 10.3389/fmicb.2024.1464719

**Published:** 2024-09-24

**Authors:** Chang Liu, Chenxuan Cui, Xiaoxin Tan, Junjie Miao, Wei Wang, Han Ren, Hua Wu, Cuiying Zheng, Huan Ren, Weijun Kang

**Affiliations:** ^1^School of Public Health, Hebei Medical University, Shijiazhuang, China; ^2^Shijiazhuang Qiaoxi Distinct Center for Disease Control and Prevention, Shijiazhuang, China; ^3^Hebei Key Laboratory of Environment and Human Health, Shijiazhuang, China; ^4^Clinical Laboratory, Xinle Traditional Chinese Medicine Hospital, Shijiazhuang, China; ^5^Clinical Laboratory, The Third Hospital of Hebei Medical University, Shijiazhuang, China

**Keywords:** *Pseudomonas aeruginosa*, gallium, pH, glutamic acid, biofilm

## Abstract

The emergence of multidrug-resistant *Pseudomonas aeruginosa* isolates is a growing concern for public health, necessitating new therapeutic strategies. Gallium nitrate [Ga(NO_3_)_3_], a medication for cancer-related hypercalcemia, has attracted great attention due to its ability to inhibit *P. aeruginosa* growth and biofilm formation by disrupting iron metabolism. However, the antibacterial efficacy of Ga(NO_3_)_3_ is not always satisfactory. It is imperative to investigate the factors that affect the bactericidal effects of Ga(NO_3_)_3_ and to identify new ways to enhance its efficacy. This study focused on the impact of pH on *P. aeruginosa* resistance to Ga(NO_3_)_3_, along with the underlying mechanism. The results indicate that acidic conditions could increase the effectiveness of Ga(NO_3_)_3_ against *P. aeruginosa* by promoting the production of pyochelin and gallium uptake. Subsequently, using glutamic acid, a clinically compatible acidic amino acid, the pH was significantly lowered and enhanced the bactericidal and inhibitory efficacy of Ga(NO_3_)_3_ against biofilm formation by *P. aeruginosa*, including a reference strain PA14 and several multidrug-resistant clinical isolates. Furthermore, we used an abscess mouse model to evaluate this combination *in vivo*; the results show that the combination of glutamic acid and Ga(NO_3_)_3_ significantly improved *P. aeruginosa* clearance. Overall, the present study demonstrates that acidic conditions can increase the sensitivity of *P. aeruginosa* to Ga(NO_3_)_3_. Combining glutamic acid and Ga(NO_3_)_3_ is a potential strategy for the treatment of *P. aeruginosa* infections.

## 1 Introduction

*Pseudomonas aeruginosa*, a Gram-negative opportunistic pathogen, causes various acute and chronic infections in patients with cystic fibrosis (*CF*), chronic obstructive pulmonary disease, and many immunocompromised patients (Rossi et al., 2021; Wood et al., 2023). It is a main cause of ventilator-associated pneumoniae (VAP), resulting in high mortality (Luyt et al., 2018; Albin et al., 2023). The coronavirus disease 2019 (COVID-19) pandemic has underscored the critical importance of treating *P. aeruginosa*-caused nosocomial VAP (Langford et al., 2020; Lansbury et al., 2020). Unfortunately, treatment of *P. aeruginosa* infections is becoming increasingly challenging due to its multiple resistant mechanisms (Subedi et al., 2018; Botelho et al., 2019; Wood et al., 2023). Current antibiotic treatments are rapidly losing their efficacy, necessitating the urgent search for new therapeutics.

A novel anti-infective approach involves exploiting the nutritional vulnerabilities of bacteria, particularly iron metabolism. Iron is incorporated into various enzymes involved in bioenergetic pathways and core carbon metabolism, making it essential for almost all pathogens, including *P. aeruginosa* (Wu et al., 2022; Schalk and Perraud, 2023). *In vivo*, free iron is scarce due to its insolubility and multiple host defenses sequestering iron (Bullen et al., 2005). Targeting iron acquisition might be an antimicrobial strategy.

One potential approach involves using metal gallium as a “Trojan horse” to disrupt the iron metabolism (Goss et al., 2018). Ga^3+^ has an identical radius to Fe^3+^, and *P. aeruginosa* iron uptake systems cannot distinguish between them, allowing Ga^3+^ to be absorbed. However, Ga^3+^ cannot be reduced in physiological conditions; its occupancy of iron sites significantly compromises iron-dependent biological functions (Wang et al., 2019). Gallium nitrate (Ga(NO_3_)_3_) is effective against *P. aeruginosa* infections. It inhibits the growth of *P. aeruginosa in vitro*, reduces the lung *P. aeruginosa* burdens, and improves survival in mice (Kaneko et al., 2007; Goss et al., 2018). Ga^3+^ is currently employed in drug development to treat *P. aeruginosa* infections in patients with *CF* (nebulized gallium citrate, Aridis Pharmaceuticals).

Recent work aimed to improve the antibacterial activity of Ga^3+^ against *P. aeruginosa* by complexing it with synthetic chelators or siderophores to stimulate Ga^3+^ uptake by the bacterium. Among various Ga^3+^-complexes including Ga-ferrichrome, Ga-dinitrate, Ga-desferrioxamine, Ga-salicylate, Ga-pyoverdine, and Ga-pyochelin, only the complex of Ga-pyochelin showed increased inhibitory activity compared to Ga(NO_3_)_3_ (Frangipani et al., 2014; Kang et al., 2021). Pyochelin (PCH), a *P. aeruginosa* siderophore, can facilitate Ga^3+^ entry into the cell through uptake machinery (Frangipani et al., 2014; Andrejević et al., 2023). PCH deficiency decreases the sensitivity of *P. aeruginosa* to Ga^3+^. Therefore, effective methods to elevate PCH production might improve the activities of Ga^3+^ against *P. aeruginosa*.

This study revealed that acidic conditions could increase Ga^3+^ sensitivity in *P. aeruginosa* by promoting PCH production and subsequently enhancing Ga^3+^ uptake. We further demonstrated that glutamic acid could decrease the pH and synergistically enhance the bactericidal effects of Ga^3+^ against *P. aeruginosa* biofilm *in vitro* as well as in a mouse abscess model. This study provides a new treatment strategy for clinical infections caused by *P. aeruginosa*.

## 2 Materials and methods

### 2.1 Strains and growth media

The lab strain used in this study was PA14 (College of Life Sciences, Nankai University). Clinical *P. aeruginosa* strains were collected from the Third Hospital of Hebei Medical University and Xinle Traditional Medical Hospital. The pEX18-*pchD* recombinant plasmid by double digestion and ClonExpress technology. PCH-deficient mutant (Δ*pchD*) was constructed through the conjugal transfer of pEX18-*pchD* and then selecting for single and double crossovers, as described previously (Schweizer, 1992).

All the strains were grown in Mueller-Hinton broth (MHB; Beijing Land Bridge). The media were acidified with HCl and alkalinized with 0.1 M NaOH. Glutamic acid (Aladdin) at 0.2% was buffered with 0.1 M Na_2_HPO_3_-KH_2_PO_3_ buffer (Sigma-Aldrich; M1442).

### 2.2 Minimum inhibitory concentrations (MICs) measurement

The MIC was determined using the broth microdilution method as per the recommendations of the Clinical and Laboratory Standards Institute (CLSI, 2024). Briefly, the highest antibiotic concentration (200 μL) was added to the first column of the 96-well plate. A volume of 100 μL of the MHB was added to Columns 2–10. A serial dilution was performed from the first to the tenth well, discarding 100 μL of the final diluted solution. Wells 11 and 12 served as negative and positive controls, respectively. Subsequently, 100 μL of the diluted bacterial suspension (1:1000) was added to the drug and the positive control columns. After 20-h incubation at 37°C, the MIC was defined as the lowest concentration without visible growth. The experiments were conducted with three biological replicates.

### 2.3 PCH production

Overnight cultures of PA14 were diluted and grown in MHB at different pH values. After 12 h, 1 mL of the culture was taken to measure PCH production, and 1 mL was taken for colony counting. The PCH was extracted using 500 μL ethyl acetate from the supernatant acidified with citric acid. PCH concentration was determined by measuring the absorbance at 320 nm (εPCH = 4,300 M^−1^ cm^−1^) (Visca et al., 1992; Porcaro et al., 2017; Visaggio et al., 2021). To eliminate the influence of pyoverdine or other extracellular pigments (such as pyocyanin) produced by *P. aeruginosa* on PCH quantification. The final PCH production was obtained by subtracting the absorbance of the Δ*pchD* mutant from the PA14 values.

### 2.4 Inductively coupled plasma mass spectrometry (ICP-MS)

One milliliter of the culture was washed twice and resuspended in 60% ultrapure nitric acid. The mixture was digested and then adjusted to a total volume of 1 mL with Milli-Q water. The gallium concentration was measured using Agilent ICP-MS 7800 (Romsang et al., 2014). The colony-forming unit of the culture was determined; the result was expressed as fmol gallium per CFU.

### 2.5 Biofilm killing assay

The overnight culture of bacteria was diluted 1:1000 with fresh MHB in 96-well plates to form the biofilm. After 24-h incubation, the planktonic bacteria were removed, and the biofilm was treated with Ga(NO_3_)_3_, with or without glutamic acid at specified concentrations. Samples were further incubated for 24 h. The plate was subsequently washed twice with normal saline for colony counting or crystal violet staining as previously described (Ren et al., 2019).

### 2.6 Biofilm inhibition assay

A 1:1000 dilution of culture was transferred into fresh MHB supplemented with different concentrations of Ga(NO_3_)_3_ and glutamic acid. A volume of 200 μL of the culture was introduced to the 96-well plate. After 24 h of incubation, the medium was discarded. The plate was cleaned with normal saline for bacteria colony counting to quantify the biofilm. Besides, the plate was washed with tap water and then stained with 0.5% crystal violet. The impact of biofilm inhibition was visually demonstrated using a mobile phone.

### 2.7 Ethic statements

Animal experiments were conducted as per US and Chinese national guidelines for the use of animals in research and were approved by the Animal Care and Use Committee of the College of Life Sciences, Nankai University.

### 2.8 Mouse cutaneous abscess model

This experiment used eight-week-old specific-pathogen-free female BALB/c mice weighing about 20 ± 2 g. Pre-experiment, mice were anesthetized using 80 μL 7.5% chloral hydrate.

For the abscess model (Pletzer et al., 2017, 2018), 50 μL of normal saline plus 4 × 10^6^ CFU of PA14 were injected subcutaneously into the right dorsum. An hour post-infection, the mice were treated with either saline, Ga(NO_3_)_3_, glutamic acid, or Ga(NO_3_)_3_-glutamic acid combinations. Three days post-infection, the mice were euthanized using carbon dioxide; the abscesses were excised, homogenized, and subjected to colony counting.

### 2.9 Statistical analysis

All the experiments were conducted at least thrice. The Student’s *t*-test was used to compare two subjects. *P < 0.05, **P < 0.01, ***P < 0.001, otherwise not significant.

## 3 Results

### 3.1 Medium acidification increases the susceptibility of *P. aeruginosa* to Ga(NO_3_)_3_

To test the role of pH in *P. aeruginosa*’s resistance to Ga^3+^, we determined the MIC of Ga(NO_3_)_3_ against the wild-type strain PA14 at different pH values. Table 1 shows that, at pH 7.4, the MIC of Ga(NO_3_)_3_ for PA14 was 200 mg/L. When the medium was alkalized to approximately 8.5 or 9.5, the MIC increased by more than fourfold. Conversely, when the medium was acidized to 6.5 or 5.5, the MIC of Ga(NO_3_)_3_ for PA14 decreased by four- or eightfold. These results suggest that the acidic conditions significantly increase the sensitivity of *P. aeruginosa* to Ga(NO_3_)_3_. Therefore, exploring a strategy to lower the pH might increase the activity of Ga(NO_3_)_3_ against *P. aeruginosa*.

**Table 1:** MIC of Ga(NO_3_)_3_ for PA14 in different pH.

pH	9.5	8.5	7.4	6.5	5.5
Ga(NO_3_)_3_ MIC (mg/L)	≥800	≥800	200	50	25

### 3.2 Glutamic acid increases the susceptibility of *P. aeruginosa* to Ga(NO_3_)_3_

To decrease the pH, we supplemented the medium with glutamic acid, an acidic amino acid. Table 2 shows that 0.1% glutamic acid can acidize the medium to a pH of 6.5 and reduce the MIC of PA14 by fourfold. The addition of 0.2% glutamic acid can acidize the pH to 5.5 and reduce the MIC by eightfold. To confirm that the effect of glutamic acid on Ga(NO_3_)_3_ sensitivity was pH-dependent, we utilized PBS buffer in the media to maintain a neutral pH. The results show that the MIC of Ga(NO_3_)_3_ was not affected by buffered glutamic acid (Table 2). Additionally, since the presence of iron in the culture medium negatively influences Ga^3+^ activity (Hijazi et al., 2018), we measured the MIC of Ga(NO_3_)_3_ for PA14 also in the iron-poor minimal medium M9. As shown in Supplementary Table S1, 0.2% glutamic acid can reduce the MIC of Ga(NO_3_)_3_ for PA14 by twofold in the M9 medium. These results indicate that glutamic acid could potentiate the effects of Ga(NO_3_)_3_ against *P. aeruginosa* through medium acidification.

**Table 2:** MIC of Ga(NO_3_)_3_ for PA14 in medium with glutamic acid.

Glutamic acid	0	0.1%	0.2%	0.1%^a^	0.2%^b^
pH	7.4	6.5	5.5	7.4	7.4
Ga(NO_3_)_3_ MIC (mg/L)	200	50	25	200	200

^a^After the addition of 0.1% glutamic acid to the medium, the pH was maintained at 7.4 through the use of PBS buffer. ^b^After the addition of 0.2% glutamic acid to the medium, the pH was maintained at 7.4 through the use of PBS buffer.

### 3.3 Acidic conditions facilitate the production of PCH and promote the uptake of Ga^3+^

**Figure 1:**
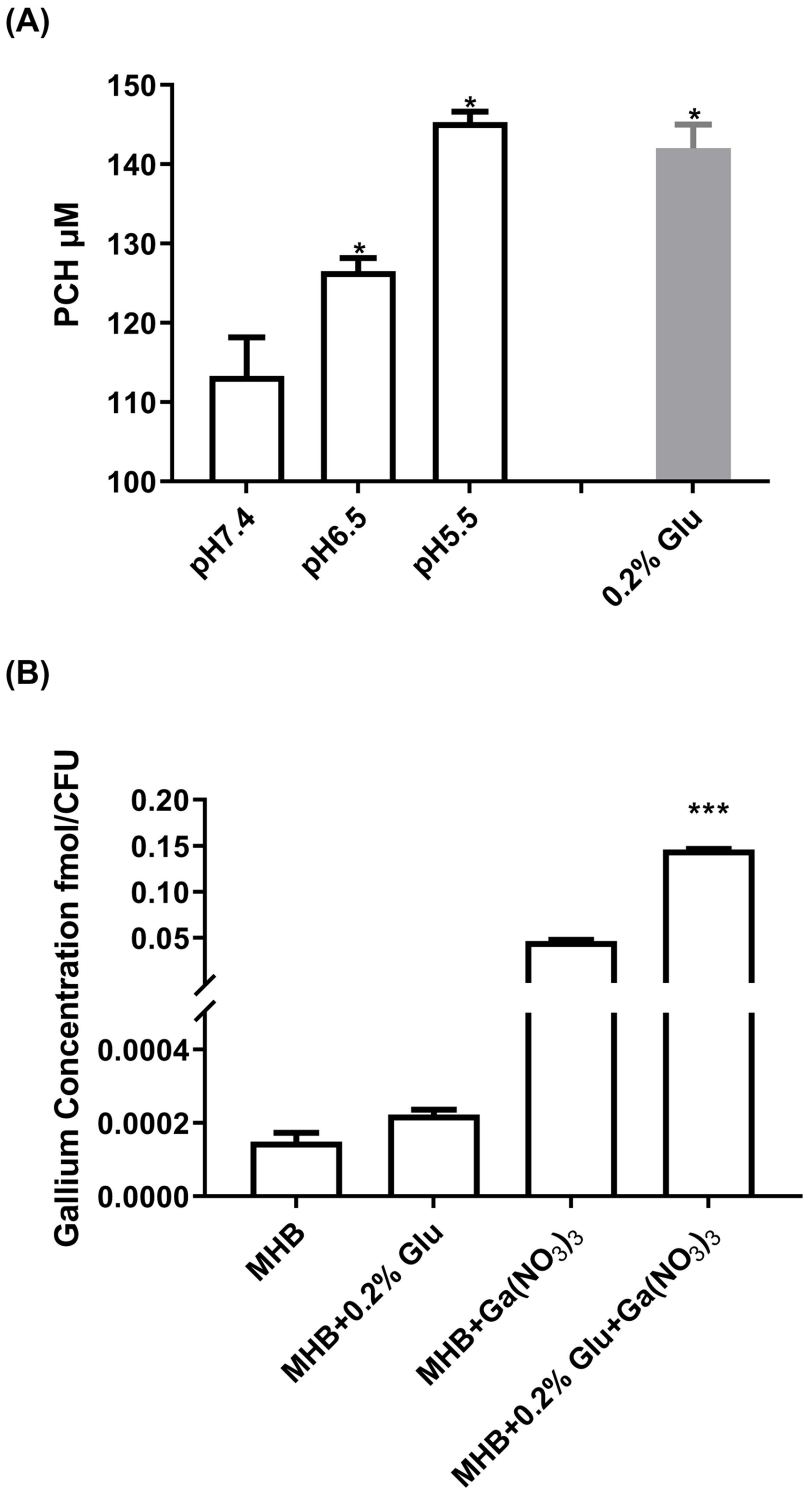
Influence of acidic conditions on PCH production and Ga^3+^ uptake by *Pseudomonas aeruginosa*. **(A)** PCH concentrations in the supernatants of the wild-type strain PA14 grown in media at pH 7.4, pH 6.5, and pH 5.5, as well as a medium treated with 0.2% glutamic acid. **(B)** The concentration of Ga absorbed in bacteria with or without 0.2% glutamic acid. To measure the influence of glutamic acid on the gallium uptake in bacteria, PA14 was treated with 25 mg/L Ga(NO_3_)_3_ in MHB with or without 0.2% glutamic acid for 12 h. Additionally, Ga(NO_3_)_3_-free medium was used as a control. The concentrations of gallium were measured using ICP-MS, and the bacterial count was determined simultaneously. Error bars represent standard deviation (*n* = 3). *p < 0.05, ***p < 0.001.

A previous study reported that the addition of PCH increased the sensitivity of *P. aeruginosa* to Ga^3+^ (Frangipani et al., 2014). To examine whether the enhanced Ga^3+^ activity under acidic conditions was due to enhanced PCH production, we first examined its production at different pH values. As shown in Figure 1A, compared to pH 7.4, the PCH secretion by *P. aeruginosa* increased by about 12 and 28% at pH 6.5 and pH 5.5; the addition of glutamic acid also induced PCH production. However, the secretion of pyoverdine (PVD), another important siderophore of *P. aeruginosa*, was not promoted in acidic conditions, and even the addition of glutamic acid decreased PVD production (Supplementary Figure S1). However, as shown in Supplementary Table S3, in the PCH knockout mutant (Δ*pchD*), the antimicrobial activity of Ga(NO_3_)_3_ did not increase under acidic conditions. These results confirm that the pH-mediated enhanced activity of Ga(NO_3_)_3_ was dependent on the PCH induction.

To further highlight the mechanism by which glutamic acid potentiates Ga^3+^ activity, we measured the intracellular Ga^3+^ levels with or without glutamic acid. Figure 1B shows that the addition of 0.2% glutamic acid promoted the uptake of Ga^3+^ by about 230%, aligning with the results of MIC and PCH production. Therefore, glutamic acid can enhance the uptake of Ga^3+^ by *P. aeruginosa*, and May become a clinical combined treatment strategy with Ga(NO_3_)_3_.

### 3.4 Glutamic acid increases the susceptibility of *Pseudomonas aeruginosa* biofilm to Ga(NO_3_)_3_

**Figure 2:**
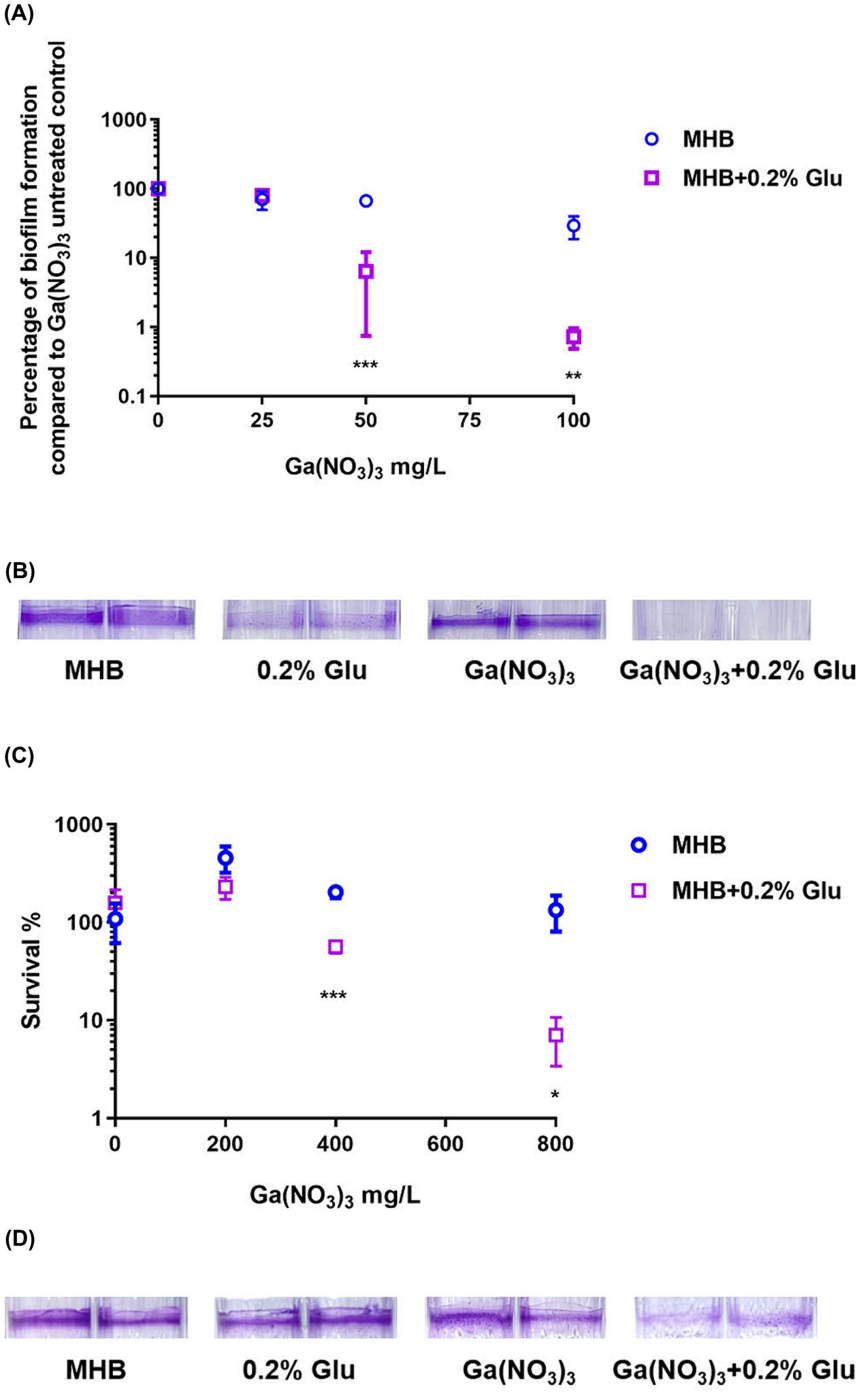
Combined effects of glutamic acid and gallium nitrate on *P. aeruginosa* biofilm. **(A)** The inhibitory effect of different concentrations (25, 50, 75, and 100 mg/L) of gallium nitrate combined with 0.2% glutamic acid on the biofilm of PA14 was measured through bacteria colony counting. **(B)** The inhibitory effect of 12.5 mg/L gallium nitrate with or without 0.2% glutamic acid on biofilm formation by PA14 was determined using crystal violet staining. **(C)** The ability of different concentrations (200, 400, 600, and 800 mg/L) of gallium nitrate combined with 0.2% glutamic acid to kill PA14 in the biofilm was detected through bacteria colony counting. **(D)** The ability of 800 mg/L of gallium nitrate with or without 0.2% glutamic acid to kill PA14 in the biofilm was determined using crystal violet staining. MHB, Mueller–Hinton broth. Glu, Glutamic acid. The mean ± SD (standard deviation) represents all values, *n* = 3. *P < 0.05, **P < 0.01, ***P < 0.001.

Biofilm formation could significantly increase antibiotic resistance in the bacterial population (Høiby et al., 2010). We thus examined the synergistic effect of glutamic acid and Ga(NO_3_)_3_ on *P. aeruginosa* biofilm. We first incubated the bacteria with Ga(NO_3_)_3_ in 0.2% glutamic acid medium to evaluate the combination effects on biofilm formation. Figures 2A,B show that using 0.2% glutamic acid or Ga(NO_3_)_3_ at 25 mg/L, 50 mg/L, or 100 mg/L had little or no effect on the formation of biofilm. Nevertheless, the combined use of Ga(NO_3_)_3_ with 0.2% glutamic acid significantly inhibited *P. aeruginosa* biofilm, as demonstrated by the bacterial CFUs (Figure 2A) and the crystal violet staining (Figure 2B).

We next examined the bactericidal effect of glutamic acid and Ga(NO_3_)_3_ on *P. aeruginosa* biofilm. Glutamic acid (at 0.2% concentration) alone could not kill the bacteria in the biofilm or eradicate the biofilm matrix. However, it decreased the survival rates of bacteria in biofilm from 457%, 203%, 109% to 230%, 56%, and 7% (Figure 2C). Crystal violet staining results also showed the same pattern (Figure 2D).

### 3.5 Synergistic effects of glutamic acid and Ga(NO_3_)_3_ against *Pseudomonas aeruginosa* clinical isolates

**Figure 3:**
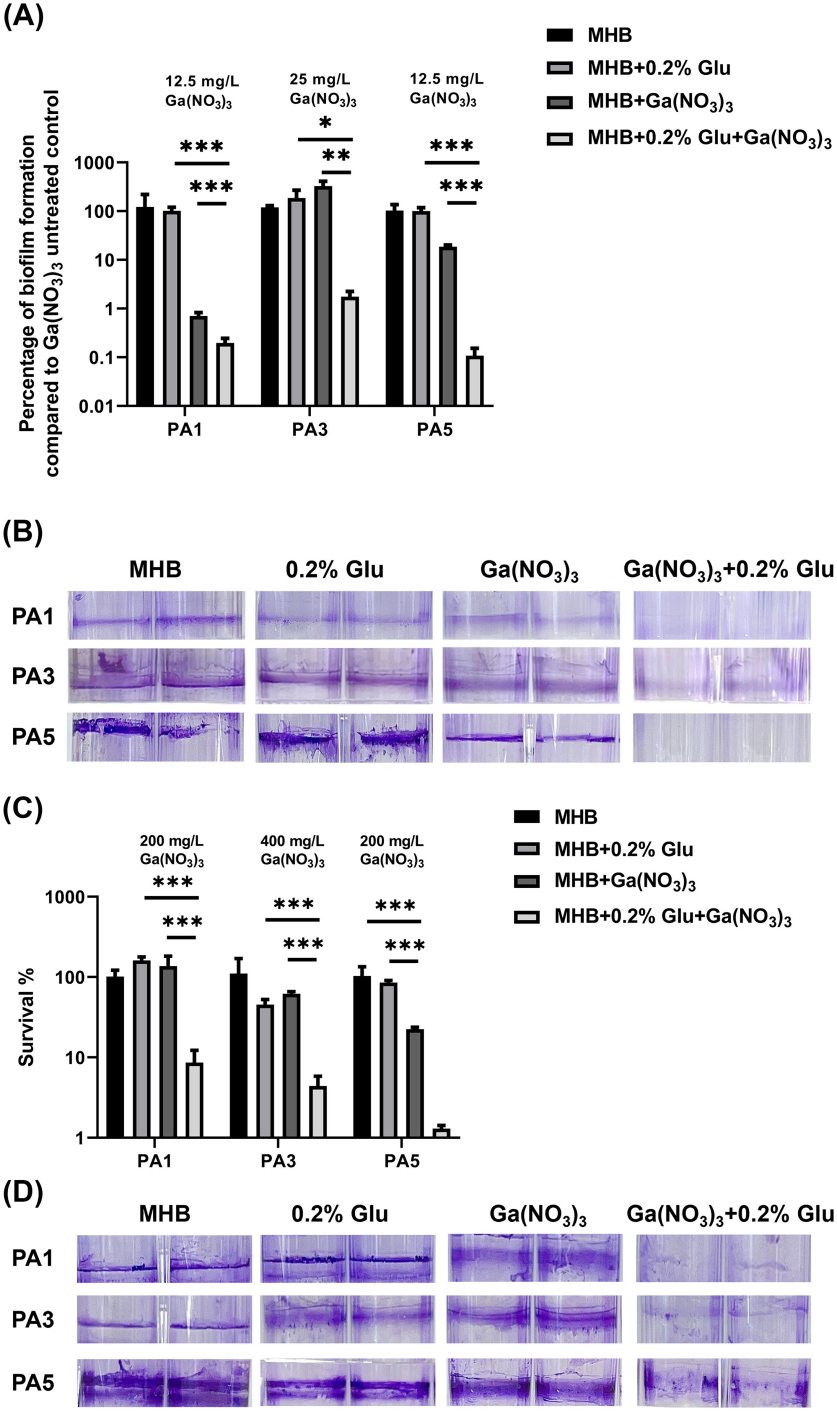
Combined effects of glutamic acid and gallium nitrate on *P. aeruginosa* clinical isolates biofilms. **(A)** PA1, PA3, and PA5 were, respectively, treated with 12.5, 25, and 12.5 mg/L of gallium nitrate combined with 0.2% glutamic acid. The inhibitory effect of these different concentrations (with or without 0.2% glutamic acid) on the biofilms of *P. aeruginosa* clinical isolates was measured through bacteria colony counting. **(B)** PA1, PA3, and PA5 were, respectively, treated with 12.5, 25, and 12.5 mg/L of gallium nitrate with or without 0.2% glutamic acid. The inhibitory effect of these concentrations (with or without 0.2% glutamic acid) on biofilm formation by these three clinical isolates was determined through crystal violet staining. **(C)** PA1, PA3, and PA5 were, respectively, treated with 200, 400, and 200 mg/L of gallium nitrate with or without 0.2% glutamic acid. The ability of these different concentrations (with or without 0.2% glutamic acid) to kill the three clinical isolates in biofilms was detected through bacteria colony counting. **(D)** PA1, PA3, and PA5 were, respectively, treated with 200, 400, and 200 mg/L of gallium nitrate with or without 0.2% glutamic acid. The ability of these different concentrations (with or without 0.2% glutamic acid) to kill the three clinical isolates in biofilms was determined through crystal violet staining. The mean ± SD (standard deviation) represents all values, *n* = 3, *P < 0.05, **P < 0.01, ***P < 0.001.

In addition to the wild-type PA14, we examined the synergistic effects of the combination against three clinical isolates. The isolation sites of the isolates and MIC of the antibiotics are listed in Supplementary Table S2. The three isolates were all multidrug-resistant bacteria. Notably, PA3 was resistant to all the antibiotics except for polymyxin. Exploring a novel therapy for these multidrug-resistant isolates is important. Herein, we measured the MIC of Ga(NO_3_)_3_ for the three isolates. The results show that the MIC of Ga(NO_3_)_3_ for the clinical isolates in MHB medium ranged from 25 to 50 mg/L (Table 3). Compared to the wild-type strain PA14, strains isolated from patients are more sensitive to Ga(NO_3_)_3_. Moreover, the addition of 0.2% glutamic acid further decreased the MIC by fourfold (Table 3). We also examined the effects of the combination on clinical isolates biofilms (Figure 3 and Supplementary Figure S2). Furthermore, the colony formation assay and crystal violet staining show that the combination of 0.2% glutamic acid and Ga(NO_3_)_3_ enhanced the inhibitory and bactericidal activities against clinical isolates, aligning with the results of PA14.

**Table 3:** MIC of Ga(NO_3_)_3_ for clinical isolates.

	PA1		PA3		PA5	
Medium	MHB	0.2% Glu*	MHB	0.2% Glu	MHB	0.2% Glu
Ga(NO_3_)_3_ MIC (mg/L)	25	6.25	50	12.5	25	6.25

*Glu, glutamic acid.

### 3.6 Combination of glutamic acid with Ga(NO_3_)_3_ synergistically improves the killing of *Pseudomonas aeruginosa* in a cutaneous abscess model

**Figure 4:**
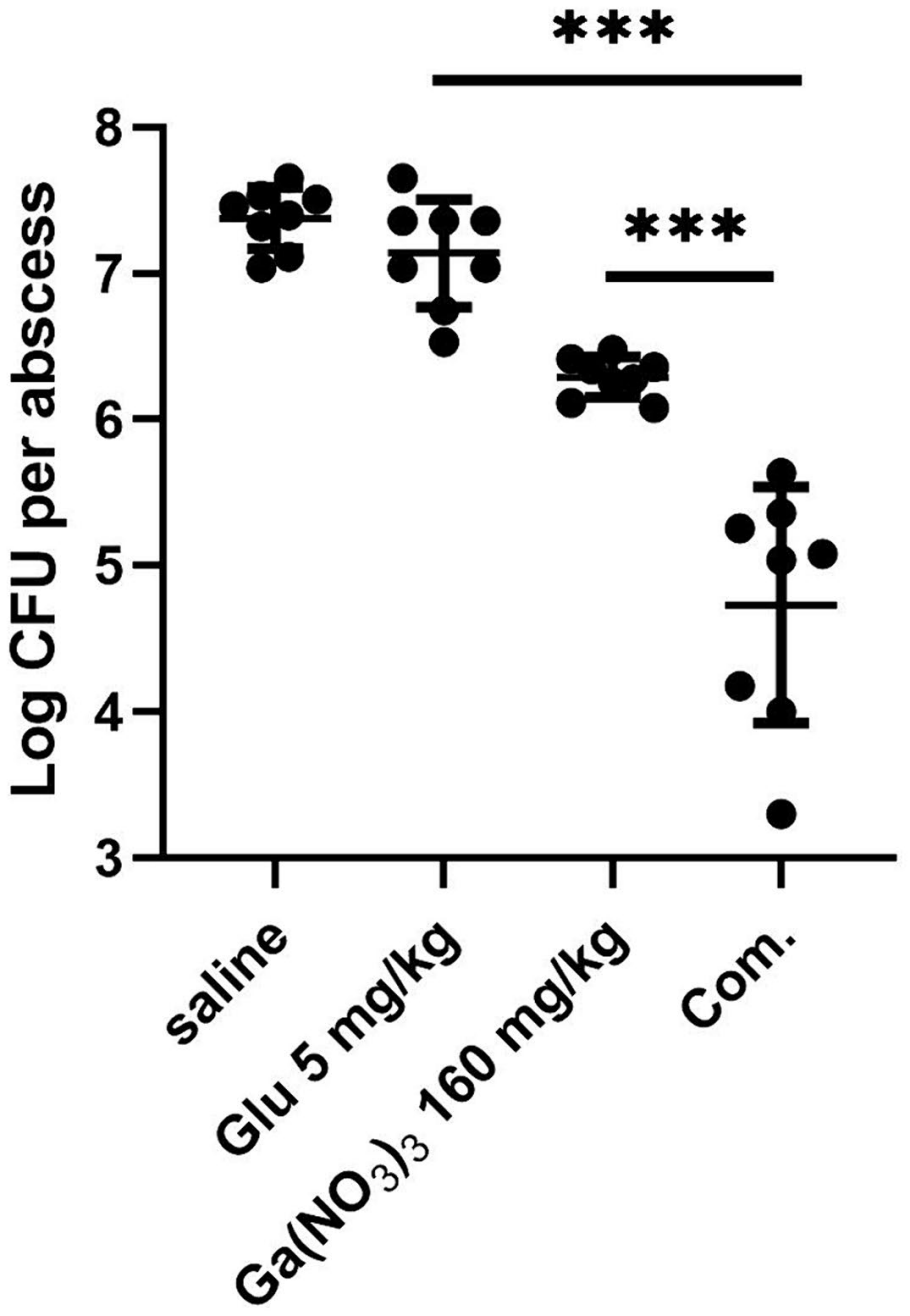
The bactericidal effect of glutamic acid combined with gallium nitrate on *P. aeruginosa* in a cutaneous abscess model. After treatment with saline, glutamic acid, gallium nitrate, and a combination of gallium nitrate and glutamic acid, the bacteria in the abscess were measured using colony counting. Com, combination. CFU, Colony-Forming Units. The mean ± SD (standard deviation) represents all values, *n* = 8, ***P < 0.001.

Encouraged by the previous *in vitro* results of the combination, we utilized a murine abscess model to test its *in vivo* efficacy of Ga(NO_3_)_3_ in combination with glutamic acid. Each mouse was infected with 4 × 10^6^ CFU PA14 and then treated with saline, glutamic acid, Ga(NO_3_)_3,_ and a combination of gallium nitrate and glutamic acid. In our assay, Ga(NO_3_)_3_ was subcutaneously injected at 160 mg/kg, and glutamic acid was administered at 5 mg/kg (2% in PBS) following previous studies (Goss et al., 2018; Jara et al., 2021). Figure 4 shows that Ga(NO_3_)_3_ and glutamic acid alone reduced the mean bacteria load by 12-fold and 2-fold over the saline control. Whereas the combination of Ga(NO_3_)_3_ and glutamic acid was highly efficacious, causing a 444-fold reduction (Figure 4). These results suggest that combining Ga(NO_3_)_3_ with glutamic acid would be a beneficial treatment for *P. aeruginosa* infection.

## 4 Discussion

Antibiotic resistance poses a significant clinical challenge in treating *P. aeruginosa* infections. Many clinical isolates of *P. aeruginosa* are susceptible to only one class of antibiotics and eventually develop pan-drug resistance. It is crucial to develop new antibiotics with good activity against multidrug resistance (MDR) *P. aeruginosa*. Gallium has been repurposed as an antibacterial agent for its capability to disrupt bacterial iron metabolism. Unlike previous antibiotics, the mechanism of gallium does not induce cross-resistance to gallium. Gallium administration in combination with other antimicrobial agents was found to be more effective than gallium administration alone against *P. aeruginosa* in several infection models (Choi et al., 2019; Scott et al., 2022). So, it’s important to investigate the factors that affect the bactericidal effects of Ga(NO_3_)_3_ and to identify combination therapy to enhance its efficacy. This study examined the effect of pH on gallium efficacy, revealing that acidic circumstances could increase the sensitivity of *P. aeruginosa* to Ga(NO_3_)_3_. Using glutamic acid as an acidic inducer increased the Ga(NO_3_)_3_ effectiveness, enhancing the eradication of *P. aeruginosa* biofilm *in vitro* and *in vivo*. As an external factor, lower pH can promote the production of bacterial virulence factors, including pyochelin, a siderophore secreted by *P. aeruginosa* (Harjai et al., 2005). Pyochelin is abundant in the extracellular environment, promoting Ga^3+^ entry into bacterial cells through a specific uptake mechanism (Braud et al., 2009; Frangipani et al., 2014). Furthermore, in acidic conditions, Ga^3+^ is more likely to exist in its free form, which enhances bacterial absorption and utilization (Zhang et al., 2023). Conversely, in alkaline solutions, Ga^3+^ mainly exists as an insoluble gallium hydroxide, limiting its bioavailability (Jensen et al., 2018). Thus, gallium nitrate, gallium maltate, and gallium citrate show significant effectiveness against bacteria (Thompson et al., 2015). Gallium competes with iron, which makes bacteria deficient in iron and affects biological processes such as electron transport, tricarboxylic acid cycle, and DNA synthesis (Olakanmi et al., 2013; García-Contreras et al., 2014; Qiao et al., 2022). Ultimately, it May make *P. aeruginosa* more sensitive to acidic environments. Thus, reducing the pH of *P. aeruginosa*’s growth environment could increase its susceptibility to gallium nitrate; however, its relevant mechanisms of regulation still need to be elucidated.

The development of new gallium-based antimicrobial combinations has broad research prospects and important clinical significance. Currently, the synthesis of new gallium coupling compounds is often used to improve its bactericidal activity, such as the newly synthesized gallium-curcumin spherical nanoparticles and gallium protoporphyrin liquid crystalline lipid nanoparticles (Awad et al., 2022; Ramesh et al., 2022). Although they May be used as effective antibacterial agents, their applications are clinically limited, and the synthesis process is relatively complex and costly. The present study used glutamic acid as an acidic inducer and combined it with gallium nitrate as a combination treatment strategy. Glutamic acid is a functional amino acid, chiefly used in psychiatric diseases; glutamate and its derivatives May be effective anticancer drugs (Murrough et al., 2017; Dutta et al., 2020). This combination significantly improved the sensitivity of *P. aeruginosa* to gallium nitrate. As a common auxiliary drug, glutamic acid is convenient to obtain without significant side effects on the body. Moreover, we found that aspartic acid can enhance the sensitivity of *P. aeruginosa* to gallium nitrate in the same way (not shown).

Importantly, this combination showed a prominent bactericidal effect in a mouse cutaneous abscess model and clinical isolates of *P. aeruginosa*, particularly the multiresistant strain PA3 (Supplementary Table S2). This strain was resistant to quinolones, carbapenems, cephalosporins, and other antibiotics except polymyxin. Furthermore, PA1 was isolated from a patient with COVID-19. Patients with COVID-19 are susceptible to co-infection with bacteria, and drug resistance is widespread, limiting medication options (Lai et al., 2021). Fortunately, glutamic acid combined with gallium nitrate could significantly inhibit and kill the PA1 and PA3 in biofilms, effectively promoting the bactericidal activity of gallium nitrate. PA1, PA3, and PA5 MDR isolates exhibited higher sensitivity to gallium nitrate compared to PA14 (four or eightfold reduction in MIC). This probably suggests a correlation between antibiotic resistance and sensitivity to gallium nitrate. Therefore, we proceeded to detect the MIC in other *P. aeruginosa* clinical isolates. Supplementary Tables S4, S5 show that these isolates exhibited diverse drug resistance profiles and demonstrated greater sensitivity to gallium nitrate than PA14 (two or fourfold reduction in MIC). Notably, the antibacterial mechanism of Ga(III) is fundamentally different from traditional antibiotics. There is no cross-resistance between antibiotics and gallium nitrate. Thus, the correlation between antibiotic resistance and gallium nitrate sensitivity has not been firmly established. Further research will conduct a more comprehensive investigation.

In summary, we have identified that acidic conditions significantly enhance the sensitivity of *P. aeruginosa* to gallium nitrate. This effect was achieved by promoting the secretion of siderophore pyochelin and the uptake of Ga^3+^ by *P. aeruginosa*. Finally, glutamic acid and gallium nitrate provided a new combination and a pharmacological treatment strategy for the clinical control and treatment of *P. aeruginosa* infection.

## Data Availability

The original contributions presented in the study are included in the article/Supplementary material, further inquiries can be directed to the corresponding authors.
